# An unusual case of adult-onset still’s disease complicated with anti-complement factor H antibodies associated atypical haemolytic uraemic syndrome

**DOI:** 10.1186/s12882-024-03548-4

**Published:** 2024-05-14

**Authors:** Winston Wing-Shing Fung, Amelia Chien-Wei Chao, Wing-Fai Pang, Raymond Siu-Ming Wong, Kai-Ming Chow, Cheuk-Chun Szeto

**Affiliations:** 1 Division of Nephrology, Department of Medicine & Therapeutics, Prince of Wales Hospital, Shatin, Hong Kong China; 2 Division of Haematology, Department of Medicine & Therapeutics, Prince of Wales Hospital, Shatin, Hong Kong China; 3 Li Ka Shing Institute of Health Sciences (LiHS), The Chinese University of Hong Kong, Shatin, Hong Kong China; 4 Department of Medicine & Therapeutics, Prince of Wales Hospital, The Chinese University of Hong Kong, Shatin, NT, Hong Kong China

**Keywords:** Atypical haemolytic syndrome, Adult-onset still?s disease, Macrophage activation syndrome, Anti-complement factor H antibodies

## Abstract

**Background:**

Atypical haemolytic uremic syndrome (aHUS) is an uncommon form of thrombotic microangiopathy (TMA). However, it remains difficult to diagnose the disease early, given its non-specific and overlapping presentation to other conditions such as thrombotic thrombocytopenic purpura and typical HUS. It is also important to identify the underlying causes and to distinguish between primary (due to a genetic abnormality leading to a dysregulated alternative complement pathway) and secondary (often attributed by severe infection or inflammation) forms of the disease, as there is now effective treatment such as monoclonal antibodies against C5 for primary aHUS. However, primary aHUS with severe inflammation are often mistaken as a secondary HUS. We presented an unusual case of adult-onset Still’s disease (AOSD) with macrophage activation syndrome (MAS), which is in fact associated with anti-complement factor H (anti-*CFH*) antibodies related aHUS. Although the aHUS may be triggered by the severe inflammation from the AOSD, the presence of anti-*CFH* antibodies suggests an underlying genetic defect in the alternative complement pathway, predisposing to primary aHUS. One should note that anti-*CFH* antibodies associated aHUS may not always associate with genetic predisposition to complement dysregulation and can be an autoimmune form of aHUS, highlighting the importance of genetic testing.

**Case presentation:**

A 42 years old man was admitted with suspected adult-onset Still’s disease. Intravenous methylprednisolone was started but patient was complicated with acute encephalopathy and low platelet. *ADAMTS13* test returned to be normal and concurrent aHUS was eventually suspected, 26 days after the initial thrombocytopenia was presented. Plasma exchange was started and patient eventually had 2 doses of eculizumab after funding was approved. Concurrent tocilizumab was also used to treat the adult-onset Still’s disease with MAS. The patient was eventually stabilised and long-term tocilizumab maintenance treatment was planned instead of eculizumab following haematology review. Although the aHUS may be a secondary event to MAS according to haematology opinion and the genetic test came back negative for the five major aHUS gene, high titre of anti-*CFH* antibodies was detected (1242 AU/ml).

**Conclusion:**

Our case highlighted the importance of prompt anti-*CFH* antibodies test and genetic testing for aHUS in patients with severe AOSD and features of TMA. Our case also emphasized testing for structural variants within the *CFH* and *CFH*-related proteins gene region, as part of the routine genetic analysis in patients with anti-*CFH* antibodies associated aHUS to improve diagnostic approaches.

## Introduction

Atypical haemolytic uremic syndrome (aHUS) is a rare, but important to recognise, form of thrombotic microangiopathy (TMA). It is characterised by a triad of microangiopathic haemolytic anaemia, thrombocytopenia, and acute kidney injury [1]. Unlike typical HUS that is associated with Shiga-toxin, aHUS is caused by genetic or acquired dysregulation of the alternative complement pathway resulting in endothelial cell dysfunction and formation of microvascular thrombi [1, 2]. It is important to recognise the underlying causes early, as there is now effective treatment for the primary aHUS. However, the distinction between the two forms can be tricky and the classification remains controversial with overlap between categories.

Anti-complement factor H (*CFH*) antibodies associated aHUS is an interesting subset of aHUS. This variant is associated with the presence of anti-*CFH* antibodies and it is previously thought to cause acquired CFH deficiency, leading to dysregulation of the complement pathway [3]. However, it is now noted the presence of anti-*CFH* antibodies is commonly associated with the deletion of the *CFH* genes, *CFHR1* and *CFHR3* [4]. However, it is a point of interest to note that not all patients with anti-factor H associated aHUS have a *CFHR1/R3* deletion and novel mutations are being studied. We present a case of adult-onset Still’s disease (AOSD) complicated by aHUS with presence of anti-*CFH* antibodies suggestive of an underlying genetic defect.

## Case report

A 42 years old man was admitted with two-week history of persistent fever, generalised maculopapular rash and a tender right cervical lymphadenopathy. He has no other respiratory, bowel or urinary symptoms. His past medical history only included eczema and there was no family history. Apart from the rash and the cervical lymphadenopathy, examination was unrevealing. Initial blood test showed a mild leucocytosis and all the other blood tests including platelet counts were unremarkable.

He was initially treated as suspected infection with viral exanthem. However, he continued to have swinging fever despite escalating antibiotics. All microbiological investigations including HIV were negative. Autoimmune screening was also unremarkable except for a raised ferritin level 8855 pmol/l. AOSD was suspected and pulsed intravenous methylprednisolone was started. Bone marrow examination showed active marrow with histiocytic proliferation, few hemophagocytic figures and no lymphoid infiltration. Cervical lymphadenopathy fine needle aspiration also showed small amount of lymphoid proliferation only, excluding lymphoma.

**Fig. 1:**
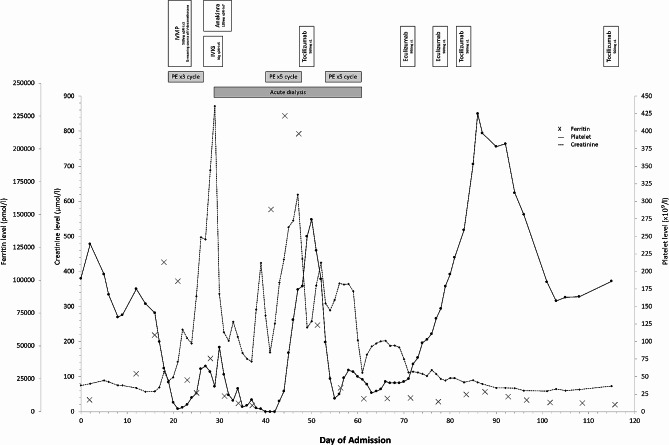
Trend of Ferritin, Creatinine and Platelet level during admission and treatments given. IVMP, Intravenous methylprednisolone; PE, plasma exchange; IVIG, intravenous immunoglobulin

Unfortunately, patient continued to deteriorate with the development of acute encephalopathy and was admitted to intensive care unit. Both computed tomography of the brain (CTB) and lumbar puncture were unremarkable. However, his platelet count started to drop to 4 × 10^9^/l (Fig. 1). INR was normal which excluded disseminated intravascular coagulopathy. Apart from the persistent inflammation from the uncontrolled sepsis and suspected AOSD, there was no other obvious triggers for the thrombocytopenia. Suspecting antibiotics was switched. Thrombotic thrombocytopenic purpura (TTP) was suspected and we were consulted to provide plasma exchange. Three cycle of plasma exchange was given until *ADAMTS13* test, taken before the start of plasma exchange, returned to be normal at 57%. Plasma exchange was thus stopped and intravenous immunoglobulin was given instead, as haematologists suspected the patient to have immune thrombocytopenia. Despite fever resolved and ferritin improved to 4700 pmol/l (peak 110,000 pmol/l), platelet only improved slightly to around 30–50 × 10^9^/l. Patient also developed progressive kidney failure with creatinine 871 µmol/l. Thus, acute haemodialysis was started (Fig. 1).

Cytokine profile CXCL9 was eventually checked and was noted to be 1120.2 pg/ml (normal 37–463 pg/ml). Natural killer cell functions were also impaired with the overall picture being compatible with macrophage activation syndrome/ hemophagocytic lymphohistiocytosis (MAS/HLH). Upon rheumatology and haematology review, anti-IL1 and dexamethasone were started, but Anti-IL1 was later stopped due to complication of fungaemia. However, patient deteriorated further and developed acute seizure with platelets dropped to 4 × 10^9^/l again. CTB was repeated and was unremarkable. Ferritin level has risen to 220,000 pmol/l and repeated cytokine profile CXCL9 was raised to 6849.0 pg/ml. Thus, flare of MAS/HLH was suspected. Haematologists was re-consulted and aHUS was suspected this time; 26 days after the initial thrombocytopenia was presented. Plasma exchange was restarted and funding for eculizumab was applied. The genetic test was performed by the Atypical hemolytic uremic syndrome (aHUS) panel (Centogene, Germany [5]). In essence, the genetic panel involved next generation sequencing (NGS) of the following main aHUS genes: *C3 convertase*, *factor H*, *factor I*, *factor B* and *membrane cofactor protein*. Multiplex ligation-dependent amplification (MLPA) was also performed to assess for structural variants within the *CFH*-*CFHR* gene region. Anti-*CFH* antibodies level was also performed at the same time by an in-house chromogenic ELISA test.

Rheumatologist also reviewed the patient and tocilizumab was started. There was significant clinical improvement following tocilizumab. Overall, the patient had 10 cycle of plasma exchange while awaiting the funding for eculizumab, which was eventually approved and two doses were given on day 71 and day 78 from the initial admission. Once the patient was stabilised, long-term tocilizumab maintenance treatment was planned instead of eculizumab following haematologist’s review. Kidney function improved gradually and patient became haemodialysis-free (Fig. 1). He was discharged to a convalescence hospital on day 116 of admission with the following salient result: Platelet 186 × 10^9^/l; Creatinine 73 µmol/l; Ferritin 5368 pmol/l. Although the aHUS may be a secondary event to flare of MAS/HLH according to haematologist’s opinion and the genetic test came back negative for the five major aHUS gene tested, high titre of anti-*CFH* antibodies was detected (1242 AU/ml). However, MLPA showed absence of *CFHR3-CFHR1* deletion. Unfortunately, the patient was lost to follow up and we were not able to have a repeated level of anti-*CFH* antibodies for comparison following treatment.

## Discussion

**Fig. 2:**
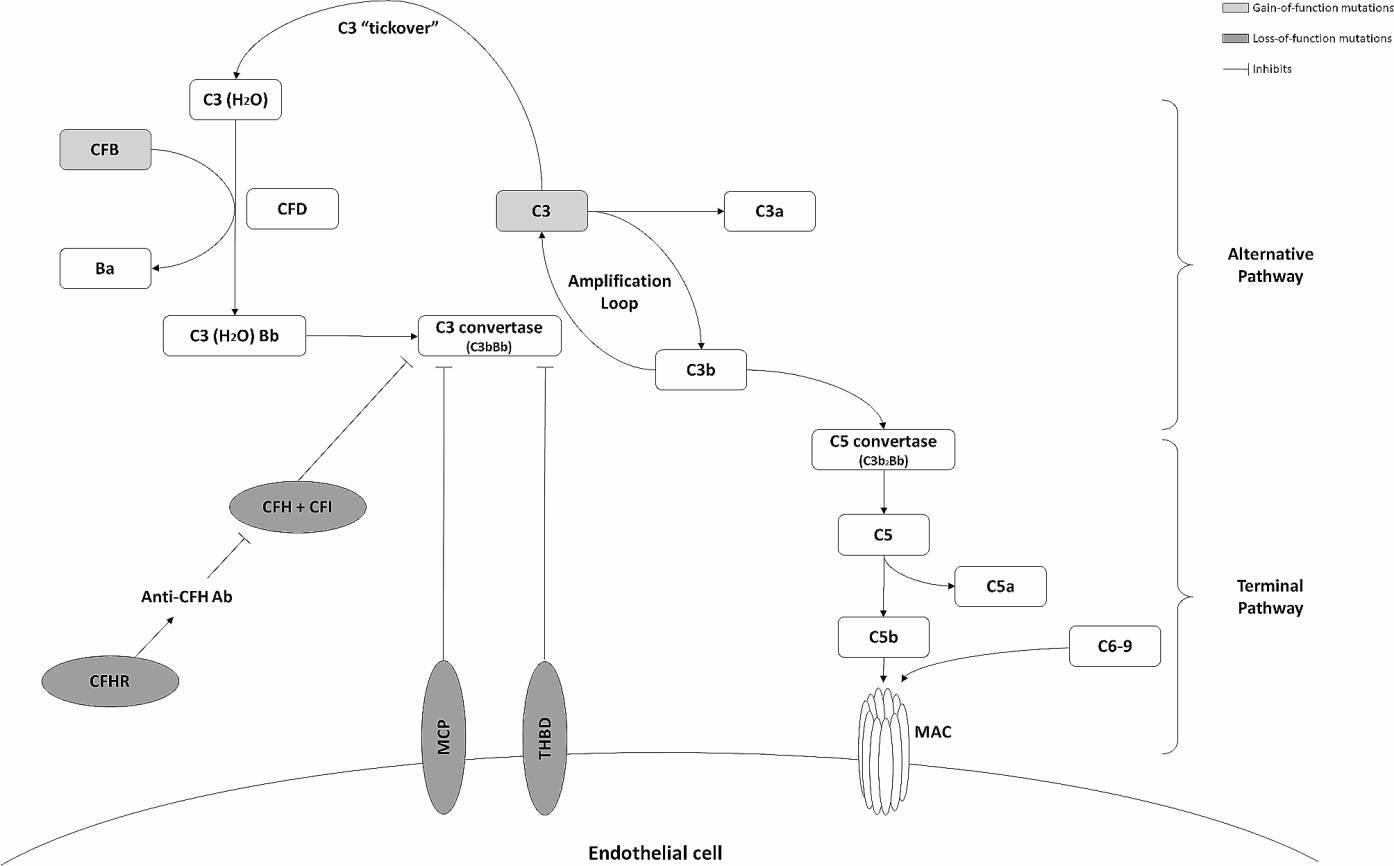
Schematic of the alternative pathway of the complement activation system and common genetic mutations in the pathway. *CFB*, complement factor B; *CFD*, complement factor D; *CFH*, complement factor H; *CFI*, complement factor I; *CFHR*, factor H-related proteins; Anti-CFH Ab, Anti-CFH antibodies; *MCP*, membrane cofactor protein; *THBD*, thrombomodulin; MAC, membrane attack complex

Our case illustrated the complexities and challenges in diagnosing aHUS, while emphasizing the importance of a high index of suspicions and early *ADAMTS13* testing to rule out TTP. In retrospect, we should consider the diagnosis of aHUS in the presence of thrombocytopenia and acute kidney injury, especially when the *ADAMTS13* came back negative. In particular, it would also be important to identify the type of aHUS. Primary aHUS mainly arises from genetic abnormalities leading to a dysregulated alternative pathway (Fig. 2); while secondary forms are attributed to a variety of condition such as severe infection, drugs and autoimmune diseases [6]. The differentiation between the two forms depends on the identification of a genetic cause or a clearly identifiable precipitating factor in the latter. However, the classification remains controversial as there is often overlap between these forms and it remains unclear whether complement dysregulation has a role in secondary aHUS [6].

Anti-*CFH* antibodies associated aHUS is a unique subgroup [3]. It occurs most commonly in paediatric cases (25–50%), although it can occur at any age (5–10%) [7, 8]. These auto-antibodies bind to the C-terminus of *CFH*, which impair the interaction of *CFH* with *C3b*. This ultimately leads to impaired regulatory function of the *CFH*, leading to dysregulation and an overactive alternative complement pathway [9]. Anti-*CFH* antibodies is not strictly acquired as several genetic mutations have been implicated in their pathogenesis. Specifically, these auto-antibodies have been shown to be strongly associated with an 80kB homozygous deletion of *CFHR3-CFHR1* in about 90% of cases with these autoantibodies [4]. This deletion is best detected by MLPA of this region [6].

Despite this, deletion of *CFHR3-CFHR1* is not a prerequisite for the formation of such autoantibodies, as there have been reports of aHUS patients with high titres of anti-*CFH* antibodies and yet no evidence of *CFHR1* or *CFHR3* mutation. It is likely that these patients have mutations in other complement genes [3, 4]. A previous study showed that mutations in *CFH*, C3, *membrane cofactor protein* (*MCP*), and *complement factor I* (*CFI*) can be associated with anti-*CFH* autoantibodies aHUS [10]. Nevertheless, it would be important to include MLPA as part of the genetic analysis, as this would enhance the diagnostic process of Anti-*CFH* antibodies associated aHUS.

Our case has also been diagnosed with AOSD complicated with MAS/HLH. AOSD is a rare systemic inflammatory disease and MAS/HLH is a life-threatening complication, characterised by excessive production of pro-inflammatory cytokines and subsequent multiple organ failure [11]. It is likely that the excessive inflammatory dysfunction could have triggered the aHUS with dysregulated complement cascade. Indeed, Khattab et al. also recently reported a similar case of AOSD associated with aHUS [12]. They did not perform genetic testing but they suggested that their case has several severe features suggestive of a *CFH* mutation. Whether this association is mainly due to a secondary aHUS with severe inflammation from AOSD or a primary aHUS with a underlying genetic defect in the complement cascade triggered by AOSD is open to debate. Interestingly, Dillemans et al. also recently presented a case of refractory AOSD accompanied by fulminant MAS/HLH and aHUS, where genetic testing revealed the patient actually had biallelic deletions in *CFHR* genes, predisposing aHUS [13].

There are implications for prompt diagnosis because there are now effective treatments. Terminal complement blockade with monoclonal antibodies against C5, such as eculizumab and ravulizumab, is considered as first-line treatment [6]. Indeed, these agents have been successfully used as induction and maintenance agent in anti-*CFH* antibodies associated aHUS [14, 15]. Plasma exchange combined with immunosuppressant have also been used as a maintenance therapy, with an aim to reduce the formation of anti-*CFH* antibodies [16]. In fact, a recent case report suggested maintenance therapy with immunosuppressants after eculizumab induction are an effective treatment [17]. However, anti-C5 monoclonal antibodies are preferred as first line, as it has less significant side effect and it has been shown to provide a better kidney recovery [15]. Further data are needed to establish optimal treatment of anti-*CFH* antibodies associated aHUS.

Treatment duration remains controversial, as there is always the risk of relapse with premature stopping. There is currently no evidence to support lifelong therapy and international consensus favoured a minimal period of treatment to allow optimal kidney recovery without early relapse [6]. Indeed, successful short-term use of eculizumab has been reported in aHUS associated with some infectious and autoimmune aetiologies [18]. Follow-up of antibody titres every 3–6 months may be helpful for monitoring patients for relapse [16]. Early relapse has been predicted by high anti-*CFH* titre (> 1500 AU/ml) [7, 19]. Interestingly, our patient is able to maintain remission so far after two doses of eculizumab and is currently on long-term tocilizumab. One possible explanation is that the tocilizumab is inducing remission for the AOSD, which is a severe proinflammatory trigger in this patient with anti-*CFH* antibodies as a predisposition for aHUS. However, as mentioned, we do not have a repeated level of anti-*CFH* antibodies to ascertain this.

In summary, our case highlighted the importance of prompt anti-*CFH* antibodies test and genetic testing for aHUS, in addition to MAS/HLH related genes, in patients with severe AOSD with MAS/HLH and features of TMA. In particular, our case also emphasized the need of MLPA to assess for structural variants within the *CFH-CFHR* gene region, as part of the routine genetic analysis for patients with aHUS and presence of anti-*CFH* antibodies to improve diagnostic and treatment approaches.

## Data Availability

All data generated or analysed during this study are included in this published article.
